# Endometriosis-Related Ovarian Cancers: Evidence for a Dichotomy in the Histogenesis of the Two Associated Histotypes

**DOI:** 10.3390/diagnostics13081425

**Published:** 2023-04-15

**Authors:** Alice Bergamini, Giorgia Mangili, Alessandro Ambrosi, Gianluca Taccagni, Emanuela Rabaiotti, Luca Bocciolone, Giorgio Candotti, Raffaella Cioffi, Francesca Pella, Giulia Sabetta, Costanza Saponaro, Massimo Candiani

**Affiliations:** 1 Obstetrics and Gynecology Unit, IRCCS San Raffaele Scientific Institute, 20132 Milan, Italy; 2 Faculty of Medicine and Surgery, Vita-Salute San Raffaele University, Via Olgettina 58, 20132 Milan, Italy; 3 Surgical Pathology Unit, IRCCS San Raffaele Scientific Institute, 20132 Milan, Italy

**Keywords:** ovarian cancer, endometrioid ovarian cancer, clear cell ovarian cancer, endometriosis, carcinogenesis

## Abstract

Evidence indicates that different pathways of malignant degeneration underlie the development of endometriosis-associated ovarian tumors of endometrioid and clear cell histotypes. The aim of this study was to compare data from patients affected by these two histotypes to investigate the hypothesis of a dichotomy in the histogenesis of these tumors. Clinical data and tumor characteristics of 48 patients who were diagnosed with either pure clear cell ovarian cancer and mixed endometrioid–clear cell ovarian cancer arising from endometriosis (ECC, *n* = 22) or endometriosis-associated endometrioid ovarian cancer (EAEOC, *n* = 26) were compared. A previous diagnosis of endometriosis was detected more frequently in the ECC group (32% vs. 4%, *p* = 0.01). The incidence of bilaterality was significantly higher in the EAOEC group (35% vs. 5%, *p* = 0.01) as well as a solid/cystic rate at gross pathology (57.7 ± 7.9% vs. 30.9 ± 7.5%, *p* = 0.02). Patients with ECC had a more advanced disease stage (41% vs. 15%; *p* = 0.04). A synchronous endometrial carcinoma was detected in 38% of EAEOC patients. A comparison of the International Federation of Gynecology and Obstetrics (FIGO) stage at diagnosis showed a significantly decreasing trend for ECC compared to EAEOC (*p* = 0.02). These findings support the hypothesis that the origin, clinical behavior and relationship with endometriosis might be different for these histotypes. ECC, unlike EAEOC, seems to develop within an endometriotic cyst, thus representing a window of possibility for ultrasound-based early diagnosis.

## 1. Introduction

Endometriosis is an estrogen-dependent chronic and progressive inflammatory disease, which affects 10% of all women of reproductive age in the world, equating to 190 million women worldwide [1,2,3]. It is characterized by the presence of extra-uterine, functionally active, endometrial tissue, represented by stroma and glands, which can be found mostly in the pelvic cavity, ovaries, fallopian tubes, sigmoid colon, appendix, upper abdomen and in other sites such as the lungs [4,5]. In particular, in 44% of cases, the ovaries are the site of endometriosis, with an endometriotic cyst defined as ovarian endometrioma [6]. The latter is evident in ultrasound examination as a unilocular cyst with homogeneous low-level echogenicity defined ground glass and absent to moderate vascularization [7]. From a molecular point of view, endometriosis is characterized by molecular abnormalities such as a loss of AT Rich Interactive Domain 1A (*ARID1A*) function, Phosphatase and Tensin homolog (*PTEN*) inactivation, Phosphatidylinositol-4,5-Bisphosphate 3-Kinase Catalytic Subunit Alpha (*PIK3CA*), Catenin Beta 1 (*CTNNB1*) and Kirsten Rat Sarcoma Viral oncogene homolog (*KRAS*) activation [6]. However, the etiology of this disease is still enigmatic. Several hypotheses have been proposed since 1870 and the most likely explanation is the retrograde menstruation theory. Additional postulated mechanisms include celomic metaplasia, lymphatic and vascular metastasis, endometrial stem cell implantation and abnormal residue of embryonic Mullerian tissue [8].

Endometriosis is a benign disease, but it shares some characteristics with cancer, such as local and distant invasion, resistance to apoptosis, recurrence, angiogenesis, damage to target organs and stimulation of the inflammatory system [9,10,11,12,13,14]. In particular, the real precursor of ovarian cancer is represented by atypical endometriosis which is seen in 60–80% of ovarian cancers that result from endometriosis [2,4].In addition, the most common mutations in atypical endometriosis affect *ARID1A*, *PIK3CA*, genes coding for estrogen and progestogen receptors, *KRAS* and *PTEN* [2].

The association between endometriosis and ovarian cancer was initially described in 1925 by Sampson, and then, it was confirmed by Scott in 1953, who observed that benign endometriosis may be present in proximity to ovarian cancer [2]. In particular, many studies confirm that the histotypes of epithelial ovarian cancer that are most closely related to endometriosis are endometrioid tumors and clear cell carcinoma [15,16]. In fact, these have mutations in common with endometriosis, such as *PTEN*, *PIK3CA*, *KRAS* and *ARID1A* [15,16,17]. 

Sarria-Santamera and colleagues highlighted that endometriosis is correlated with a 2.66-fold greater risk of ovarian cancer, compared to the general population [18]. In addition, it has been estimated that the lifetime risk of ovarian cancer in women with endometriosis is 2.5% [1].

In a recent systematic review and meta-analysis by Kvaskoff and collaborators, it was estimated that those with clear cell and endometrioid histotypes had a greater risk of endometriosis, equal to 3.4-fold and 2.3-fold, respectively [9]. In particular, endometriosis is observed in 21–51% of women with clear cell ovarian cancer (CC) (odds ratio OR = 3.05) and in 23–43% of patients with endometrioid ovarian cancer (EOC) (OR = 2.04) [19]. Therefore, about one-third of all endometrioid and clear cell histotypes are estimated to arise from endometriosis; however, the mechanisms underlying the cancerogenesis of each subtype are not completely clear [19]. There is an increasing body of evidence suggesting that clear cell histotypes may arise from pre-existing endometriosis derived from retrograde menstruation, while endometrioid cancer derived from ovarian Mullerian metaplasia [20], with different pathways of malignant degeneration and different precursors, might be involved in the development of the two endometriosis-associated histotypes. In a very interesting paper, Kajihara and coworkers hypothesized a potential dichotomy in their histogenesis, suggesting that clear cell ovarian cancers which are associated with endometriosis may arise from pre-existing endometriosis derived from retrograde menstruation, whereas ovarian Mullerian metaplasia might be the initial event in the development of endometriosis-associated endometrioid cancers. Very few clinical studies have provided evidence forthe theory that for these two histotypes, the clinical behavior, prognosis and, most importantly, the origin and relation to endometriosis, might be different [20]. To the best of our knowledge, there are no studies which have specifically assessed this issue. The main reason is that, in several clinical series, these two histologies are often considered as a single entity, which are referred to as “endometriosis-associated ovarian tumors” [6]. This, together with the lack of adherence to the criteria for the pathological diagnosis of endometriosis-associated endometrioid ovarian tumors, as originally described by Sampson and Scott [21,22], might have prevented the correct identification of the study population. Finally, clinical studies addressing this area have, in general, compared characteristics of histotype-specific tumors which are associated, or are not associated, with endometriosis and not between endometriosis-associated cancers of different histotypes [9,23].

As a matter of fact, in two previous studies, our group separately analyzed these two histotypes, comparing endometrioid and clear cell carcinomas which were associated, or were not associated, with endometriosis [24,25]. In the first study considering endometrioid histology, we showed that compared to patients without endometriosis, women with endometriosis-associated endometrioid ovarian cancer were significantly younger at diagnosis, had a lower disease stage and had a less prevalent high-grade tumor. Interestingly, the rate of synchronous endometrial cancer was significantly higher in this group [24]. In the second study considering clear cell histology, patients with tumors arising from endometriosis were significantly younger, more frequently had a unilateral involvement and had a lower prevalence of ascites as the presenting symptom. No difference between the two groups was found for the International Federation of Gynecology and Obstetrics (FIGO) stage, laterality or the presence of a synchronous endometrial malignancy [25].

The aim of the present study was to clinically compare clinical data and tumor characteristics of patients affected by ovarian tumors of different histotypes which were associated with endometriosis, in order to verify the hypothesis supporting a dichotomy in the histogenesis of endometriosis-associated ovarian carcinomas, thus potentially offering interesting and novel clinical insights regarding this issue.

## 2. Materials and Methods

**Figure 1:**
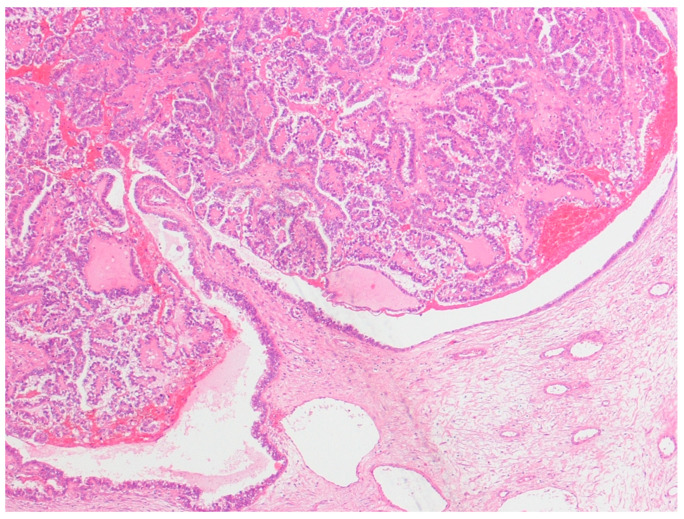
Clear cell carcinoma of the ovary arising in the lumen of an endometrioma, lined by columnar endometrioid cells. Hematoxylin and eosin × 150.

This was a retrospective study of 48 cases of ovarian tumors strictly associated with endometriosis, which were diagnosed and consecutively treated at the Obstetrics and Gynecology Unit of the Scientific Institute San Raffaele in Milan, Italy, between 1995 and 2016. Ethical approval was obtained from the San Raffaele Institute Ethics Board. All patients with a primary diagnosis of either pure clear cell ovarian cancer and mixed endometrioid–clear cell ovarian cancer strictly arising from endometriosis (ECC), or with endometriosis-associated endometrioid ovarian cancer (EAEOC), were included in the study. Patients whose diagnosis was made elsewhere were excluded. The definition of endometriosis arising from ovarian cancer was given according to Sampson’s [21] and Scott’s criteria [22], which included: (1) the coexistence of carcinoma and endometriosis in the same ovary; (2) the presence of tissue similar to endometrial stroma surrounding characteristic epithelial glands; (3) the exclusion of a metastatic tumor to the ovary and (4) the presence of benign endometriosis histologically contiguous with the malignant tissue. Patients with clear cell carcinoma associated with, but not arising from, endometriosis were excluded (Figure 1).

All patients underwent surgery, received chemotherapy and were followed up at our institution. Surgical staging was performed according to FIGO guidelines for ovarian cancer, including total abdominal hysterectomy, bilateral salpingo-oophorectomy, omentectomy and the removal of all macroscopic diseases [26]. All pathological analysis was performed by the same gynecologic pathologist.

The patients were divided into two groups according to histology (EAEOC or ECC). Data including age at diagnosis, clinical presentation, history, disease status and pathological information, such as histology, stage, laterality, presence of concurrent endometrial carcinoma and macroscopic appearance of the tumor at surgery, were collected from surgical and pathology reports. The macroscopic appearance of the tumor was reported as the rate between the solid and cystic components at gross histology, expressed as a percentage. Stages higher than IIA were classified as advanced, while lower stages were considered early. The history of endometriosis, accounting for either previous surgery for endometriosis, or the ultrasound detection of an endometriotic cyst, was indicated. All the above-mentioned variables were described for each of the two groups and statistically compared. Statistical analyses were performed using Statistical Package for Social Science (SPSS) version 28.0 (SPSS Inc., Chicago, IL, USA) and the R environment. The Pearson chi-square test or the Student’s t-test were used to assess the significance of differences in clinical and pathological variables between the two groups. We also investigated the difference in FIGO stage across time points between ECC and EAEOC by means of the regression model “*stage* = (α_0 EAEOC_ + *I_ECC_* α_0_) + (α_1 EAEOC_ + *I_ECC_* α_1_) *time*” where “α_0 EAEOC_” and “α_1 EAEOC_” are the intercept and the slope of the model for EAEOC patients, respectively; “*I_ECC_*” is the dummy variable for ECC patients and “α_0_” and “α_1_” are the intercepts difference and the slopes difference between EAEOC and ECC patients, respectively. In all analyses, a *p* value of <0.05 was considered statistically significant.

## 3. Results

Medical records and pathologic specimens were available for *n* = 48 patients diagnosed with endometriosis-associated ovarian cancer in the considered time interval. Of these, *n* = 26 (54%) had EAEOC and *n* = 22 (46%) had ECC. The clinical and morphological characteristics of the two groups are shown in Table 1. Age was not significantly different between the two groups. Interestingly, seven patients in the ECC group (32%) were previously diagnosed or operated on for endometriosis, while only one patient (4%) in the EAEOC group reported a history of endometriosis (*p* = 0.01). Considering the clinical presentation, the symptoms did not differ statistically between the two groups, except for abdominal pain, which was significantly more frequent in the EAEOC group (46% vs. 14%, *p* = 0.02) (Table 1).

As shown in Table 1, 85% of EAEOC subjects were diagnosed with early-stage disease, in contrast to 59% of the ECC subjects, as the FIGO stages were significantly different between the two groups (*p* = 0.04). The incidence of bilaterality was significantly higher in the EAOEC group as compared to the ECC group (35% vs. 5%, *p* = 0.01). Additionally, considering the macroscopic appearance of the tumor, the solid/cystic rate at histological examination was significantly higher for the EAOEC group (57.7 ± 7.9% vs. 30.9 ± 7.5%, *p* = 0.02). Interestingly, 10 patients in the EAEOC group (38%) had a diagnosis of synchronous endometrial cancer while none of the patients in the ECC group did (*p* = 0.001).

**Figure 2:**
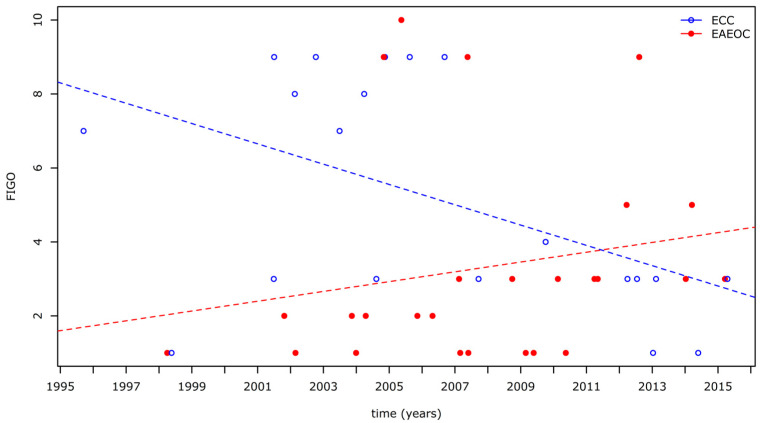
Trends of FIGO stage at diagnosis according to the year of detection for EAEOC and ECC.

Trends in FIGO stage at diagnosis, as a function of the year of detection, are reported in Figure 2 in relation to the specific histotype. According to the multivariate regression analysis, the year of diagnosis was a significant predictor of FIGO stage for the clear cell histotype (*p* = 0.02) while it was not for the EAEOC histotype (*p* = 0.32; slopes difference: *p* = 0.02).

## 4. Discussion

The malignant transformation of endometriosis is, in general, a rare event that occurs in between 0.7–1.6% of women [27].

In a recent systematic review and meta-analysis by Kvaskoff and collaborators, it has been estimated that endometriosis is associated with a greater risk of clear cell and endometrioid histotypes, equal to 3.4-fold and 2.3-fold, respectively [9]. In particular, these authors pooled the results of 24 studies (case–control and cohort studies), published between January 1990 and January 2020, on the relationship between endometriosis and ovarian cancer, and investigat the impact of endometriosis on the risk and prognosis of ovarian cancer (summary relative risk—[SRR] = 1.93, 95% confidential interval—[CI] = 1.68–2.22). A stronger association with endometriosis was found for the clear cell histotype (SRR = 3.44, 95% CI = 2.82–4.20) while a still significant but weaker association was reported for the endometrioid histotype (SRR = 2.33, 95% CI = 1.82–2.98) [9].

The carcinogenic pathways underlying the malignant transformation of endometriosis are not completely clear. Endometriosis-associated ovarian carcinogenesis is a multistep process, in which a precursor lesion, such as atypical endometriosis, harboring key mutations, progressively accumulates genetic and epigenetic changes, which are promoted by the inflammatory, hyperestrogenic environment and oxidative milieu of the endometriotic lesion [2].

In particular, it has been proposed that the transition from endometrioma to atypical endometriosis may be caused by oxidative stress, due to reactive oxygen species developing within the blood, which are present in endometriomas. This causes genetic mutations, initially affecting the oncosuppressor gene *ARID1A* and subsequently affecting *PIK3CA*. The accumulation of these genetic alterations induces the transition from atypical endometriosis to ovarian cancer [2].

There is, however, an increasing body of evidence from clinicopathological studies suggesting that separate pathways of malignant degeneration of endometriosis might be involved in clear cell and endometrioid tumors and that their relationship with endometriosis might be different [19]. In particular, atypical endometriosis, meaning cytological and histological atypia such as a hyperchromatic nucleus, an increased nucleus-cytoplasm ratio and cell crowding, is considered to be a direct precursor of epithelial ovarian cancer. In fact, it is present in 60–80% of tumors associated with endometriosis, but it is more frequently associated with clear cells tumors (36%), compared with endometrioid cancer (23%) [4]. Furthermore, the trigger of carcinogenesis in clear cell carcinomas may be due to oxidative stress generated by iron-related substances, due to the repeated hemorrhages that occur in endometriosis, while for endometrioid cancer, Müllerian metaplasia has been considered to be the main mechanism involved [4].

From a biological standpoint, support for this hypothesis derives from the investigation of the hepatocyte nuclear factor (HNF-1β) overexpression in clear cell carcinoma of the ovary associated with endometriosis. In fact, from an immunohistochemical point of view, this factor is only detected in eutopic and ectopic endometrium and in clear-cell-type tumors. This supports the theory of endometriosis expressing HNF-1β as the precursor of endometriosis-associated clear cell carcinoma [20]. Conversely, the absence of HNF-1β expression in endometrioid histology and in ovarian cortical inclusion cysts would support the metaplastic transformation of the inclusion cysts into a Müllerian epithelium as a precursor of endometrioid tumor development [20]. In addition, ovarian cancer associated with endometriosis is correlated with the same molecular features that are present in endometriosis (particularly in the atypical variant) and type 1 ovarian cancer, such as PIK3CA and *KRAS* activating mutations and *ARID1A* and *PTEN* inactivating mutations, although there are differences in the frequency of these alterations between the two subtypes of ovarian cancer. *ARID1A* mutations are found in 46% of ECC and 30% of EAEOC, PIK3CA mutations are present in 41–57% of ECC and in 30–48% of EAEOC and finally, PTEN is mutated in 25% of ECC and in 20% of EAEOC. Moreover, *KRAS* and *CTNNB1* are mutated in 29% and 40% of EAEOC, respectively, and in 7% and 3% of ECC, respectively [6,27]. From a clinical standpoint, very few studies have highlighted the different behavior of these two histotypes with respect to endometriosis.

**Figure 3:**
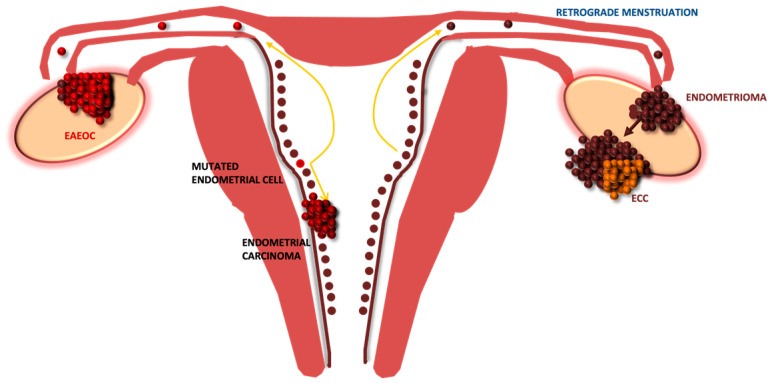
Dichotomy in the histogenesis of endometriosis-associated ovarian cancer histotypes. In EAEOC, the original precursor might derive from the endometrium, while evidence would support ECC carcinogenesis to occur within the endometrioma. EAEOC: endometriosis-associated endometrioid ovarian carcinoma; ECC: endometriosis-associated clear cell ovarian carcinoma.

Indeed, we agree on the existence of a dichotomy in the etiology of the two different ovarian tumors correlated with endometriosis. However, evidence from our previous studies and the common molecular alterations found to be shared between endometriosis-associated endometrioid ovarian cancer and type I endometrial carcinoma has led us to postulate some different novel hypotheses, suggesting a parallelism between endometrial and ovarian endometrioid tumors [24,25]. It is known that the association between endometrial neoplasm and ovarian endometrioid cancer occurs in 3.1–10.0% of patients with endometrial cancer and in 10% of those with ovarian cancer [28]. In fact, in a recent retrospective cohort study by Ishizaka and colleagues has demonstrated that endometrial cancer associated with endometriosis has a high probability of being simultaneous at ovarian carcinoma, in particular, endometrioid histotype is the most common histological subtype present [29]. Moreover, different types of gene-based biomarkers such as *ARID1A*, *PIK3CA*, *KRAS* and *CTNNB1* are recurrently mutated in endometrial cancer type I, endometriosis and endometriosis-associated ovarian tumors [30]. According to this idea, which is summarized in Figure 3, the original precursor of EAEOC might be found in the endometrium, where an already mutated endometrial cell might lead, via retrograde menstruation, to the development of ovarian endometriosis.

Interestingly, in the current series, we confirmed the detection of synchronous endometrial carcinoma only in EAEOC and not in ECC. Conversely, the endometriotic cyst, which is often detected several years before, might be the setting for the development of clear cell carcinomas, but not for endometrioid ovarian cancer. In fact, the carcinogenesis underlying the transformation of endometriosis to clear cell carcinoma would be different: it might originate from and slowly progress within the endometriotic cyst, due to the effect of oxidative stress and inflammation, which involves different pathways [2,3,6]. This hypothesis for a dichotomy in the etiology of the two histotypes finds further support in the current analysis. Firstly, a history of known endometriosis was reported more frequently in the ECC group than the EAEOC group, with a statistically significant difference. Moreover, the cystic nature of clear cell carcinoma might be explained by the significantly higher detection of a unilateral lesion in the ECC group, considering that endometriomas are rarely bilateral. In line with this observation, the solid/cystic rate in the ECC group at macroscopic histology was significantly lower than the EAEOC group. Arguments against the theory by Kajihara et al. include (i) the demonstration by various authors of the endometrioid histology in synchronous ovarian and endometrial carcinomas associated with endometriosis which is not consistent with the endometrioid tumor development from the Müllerian epithelium [29,30]; (ii) evidence supporting different origins for the various forms of endometriosis [8] does not tend to indicate two different histogeneses for the endometriotic cyst, as the theory of Kajihara and coworkers would imply. Endometriosis in different sites might indeed have a different histogenesis. On the other hand, the possibility that clear cell carcinoma cancer might originate from pre-existing endometriosis, derived from regurgitated endometrial cells on the ovarian surface, contrasts with the observation that in 61% of ovarian carcinomas, which develop secondary to pre-existing, benign-appearing endometriomas which have a long latency interval (mean 4.5 years) between the benign and the malign entities, the histologic findings are clear cell carcinoma [27,31].

It is important to consider that if the theory of slow development of clear cell carcinoma within an already known endometrioma is true, then we would expect ultrasound monitoring to be effective only in the early detection of ECC, but not EAEOC. As a matter of fact, in our study population, between 1995 and 2016, a significantly decreasing trend in the FIGO stage at diagnosis was identified for ECC, but not for EAEOC, and the difference between the trends was statistically significant (*p* = 0.02). This could find a possible explanation in the diffusion of ultrasound as a follow-up technology for ovarian endometriotic cysts and in the effort of some authors to study and describe the peculiar and early sonographic appearance of the malignant degeneration of endometriomas [7,32,33].

In particular, from an ultrasonographic standpoint, endometriosis-associated endometrioid carcinomas are mainly unilateral, unilocular-solid and often contain papillary projections. Conversely, the typical ultrasound pattern of endometrioid ovarian carcinomas not developing from endometriosis is cockade-like, which refers to a cyst with a large central solid component entrapped within locules [32]. Clear cell tumors do not show a typical pattern, as this type of tumor more often shows a ground-glass echogenicity of cyst fluid [33].

The clinical implications of this finding are important considering that clear cell carcinoma is known for its low chemosensitivity and poor prognosis in advanced-stage disease, while the survival rate of adequately staged IA disease is greater than 95% [34,35]. The stage at diagnosis of clear cell carcinomas arising from endometriosis is expected to further decrease with the increasing use of ultrasound in the follow-up of endometriotic cysts, thus allowing clinicians to identify a subset of patients with a better prognosis. Moreover, a meta-analysis by Chen and colleagues highlighted better overall survival (OS) and progression-free survival (PSF) in patients with endometriosis-associated ovarian cancer compared to women with neoplasms not related to endometriosis [10]. More studies are needed to understand if there is a difference in terms of OS and PSF between clear cell carcinoma and endometrioid carcinoma associated with endometriosis.

Our study has several limitations, with the main limitation being its small sample size; these results should be interpreted with caution and need to be confirmed in larger cohorts. The next step of this study could involve the separate investigation of clear cell and endometrioid ovarian tumors with and without associated endometriosis. This more complete design and the simultaneous increase in the number of cases will be helpful in better defining the differences in terms of clinical behavior, outcomes and prognosis between these two different entities. Another limitation is the lack of molecular characterization of these tumors, which could be helpful in understanding the biology underlying carcinogenesis and its relationship with endometriosis.

## 5. Conclusions

Among endometriosis-associated ovarian cancers, ECC, unlike EAEOC, seems to be characterized by slow development within an endometriotic cyst, thus representing a subset of diseases where ultrasound could be effective in the early detection of malignant degeneration. However, even if the clinical evidence suggests that there is a dichotomy in the etiology of endometriosis-associated ovarian cancer, the biology underlying carcinogenesis still remains unclear and this should therefore be addressed by further larger studies.

## Figures and Tables

**Table 1:** Clinical and pathological characteristics of endometriosis-associated ovarian tumors.

Characteristics	EAEOC (*n* = 26)	ECC (*n* = 22)	*p*
Age (mean ± SD)	53.4 ± 9.1	53.1 ± 10.1	0.9
Symptoms			
Abdominal pain	12 (46%)	3 (14%)	**0.02**
Abdominal distension	11 (42%)	5 (23%)	0.13
Vaginal bleeding	7 (27%)	2 (9%)	0.11
Fatigue	1 (4%)	1 (5%)	0.70
Incidental diagnosis	6 (23%)	4 (18%)	0.54
Ascites	3 (11%)	0	0.15
History of endometriosis	1 (4%)	7 (32%)	**0.01**
FIGO stage			**0.04**
Early stage	22 (85%)	13 (59%)	
Advanced stage	4 (15%)	9 (41%)	
Grading			**<0.001**
1	10 (40%)	0	
2	8 (30%)	0	
3	8 (30%)	22 (100%)	
Tumor side			**0.01**
Monolateral	17 (65%)	21 (95%)	
Bilateral involvement	9 (35%)	1 (5%)	
Solid/cystic tumor rate (%)	57.7 ± 7.9	30.9 ± 7.5	**0.02**
Synchronous endometrial cancer	10	0	**0.001**

Bold: Means statistically significant (*p* < 0.05).

## Data Availability

Data are stored at the Obstetrics and Gynecology Unit, San Raffaele Hospital, Milan, Italy.
